# 5-Hydroxyethyl-3-tetradecanoyltetramic acid represents a novel treatment for intravascular catheter infections due to *Staphylococcus aureus*

**DOI:** 10.1093/jac/dkw482

**Published:** 2016-12-19

**Authors:** Marta Zapotoczna, Ewan J. Murray, Siobhan Hogan, James P. O’Gara, Siri R. Chhabra, Weng C. Chan, Eoghan O’Neill, Paul Williams

**Affiliations:** 1 Department of Clinical Microbiology, Royal College of Surgeons in Ireland, Beaumont Hospital, Dublin, Ireland; 2 School of Life Sciences, Centre for Biomolecular Sciences, University of Nottingham, University Park, Nottingham, UK; 3 Department of Microbiology, School of Natural Sciences, National University of Ireland, Galway, Ireland; 4 School of Pharmacy, Centre for Biomolecular Sciences, University of Nottingham, University Park, Nottingham, UK; 5 Department of Microbiology, Connolly Hospital, Dublin, Ireland

## Abstract

**Objectives:** Biofilm infections of intravascular catheters caused by *Staphylococcus aureus* may be treated with catheter lock solutions (CLSs). Here we investigated the antibacterial activity, cytotoxicity and CLS potential of 5-hydroxyethyl-3-tetradecanoyltetramic acid (5HE-C14-TMA) compared with the related compounds 3-tetradecanoyltetronic (C14-TOA) and 3-tetradecanoylthiotetronic (C14-TTA), which are variants of quorum sensing signalling molecules produced by *Pseudomonas aeruginosa*.

**Methods:** Antibacterial activity and mechanism of action of 5HE-C14-TMA, C14-TOA and C14-TTA were determined via MIC, bacterial killing, membrane potential and permeability assays. Susceptibility of *S. aureus* biofilms formed in the presence of plasma *in vitro* was investigated, MTT cytotoxicity testing was undertaken and cytokine release in human blood upon exposure to 5HE-C14-TMA and/or *S. aureus* biofilms was quantified. The effectiveness of 5HE-C14-TMA as CLS therapy *in vivo* was assessed using a rat intravascular catheter biofilm infection model.

**Results:** MICs of 5HE-C14-TMA, C14-TOA and C14-TTA ranged from 2 to 4 mg/L. 5HE-C14-TMA and C14-TTA were bactericidal; all three compounds perturbed the staphylococcal membrane by increasing membrane permeability, depolarized the transmembrane potential and caused ATP leakage. Cytotoxicity and haemolytic activity were compound and target cell type-dependent. 5HE-C14-TMA reduced *S. aureus* biofilm viability in a dose-dependent manner *in vitro* and *in vivo* and did not trigger release of cytokines in human blood, but inhibited the high levels of IL-8 and TNF-α induced by *S. aureus* biofilms.

**Conclusions:** 5HE-C14-TMA, C14-TOA and C14-TTA are membrane-active agents. 5HE-C14-TMA was the most potent, eradicating *S. aureus* biofilms at 512–1024 mg/L both *in vitro* and *in vivo* as a CLS.

## Introduction

In routine healthcare, use of implantable medical devices, such as intravascular catheters (IVC), has increased significantly. However, colonization by surface-adhering bacteria is associated with biofilm formation and subsequent catheter-related bloodstream infection (CRBSI) resulting in significant patient morbidity and mortality, prolonged hospitalization and excess hospital-related costs.^1^^,^^2^ Staphylococcal biofilms are recognized as the most frequent cause of CRBSI.^3^^,^^4^ Such biofilms are highly refractory to both the innate immune system and antimicrobial therapy resulting in treatment failure and persistence of infection.

While the presence of a *Staphylococcus aureus* CRBSI usually necessitates systemic antimicrobial therapy along with IVC removal, there are common clinical situations that preclude IVC removal such as the lack of alternative vascular access, patient co-morbidities and bleeding disorders. An alternative treatment approach attempting to salvage the IVC and eradicate the biofilm, involves the combination of systemic antimicrobials and a catheter lock solution (CLS) filling the lumen of the IVC to deliver high concentrations of an antimicrobial at the site of infection. With respect to staphylococcal IVC infections, the Infectious Disease Society of America guidelines on CRBSI management recommend the use of CLSs for IVC salvage.^5^ However, there is no consensus on the most appropriate agent for use as a CLS. Several commonly used antibiotics and antiseptics are ineffective in the treatment of *S. aureus* biofilm infections.^6^

Given the increasing use of medical devices and the global threat posed by multi-antibiotic-resistant bacteria there is renewed interest in novel antimicrobials that inhibit bacterial growth or control infection through attenuation of virulence and biofilm formation.^7^ During a search for novel chemical scaffolds for such compounds, we discovered that the *Pseudomonas aeruginosa* quorum sensing signal molecule, *N*-(3-oxododecanoyl)-L-homoserine lactone (3-oxo-C12-HSL) inhibited *S. aureus* growth at high concentrations (MIC, 100 μM; 30 mg/L).^8^^,^^9^ At subinhibitory concentrations, 3-oxo-C12-HSL inhibited production of *S. aureus* exotoxin virulence determinants, including α-haemolysin and toxic shock syndrome toxin by antagonizing *agr*-dependent quorum sensing probably via an allosteric interaction with the sensor kinase AgrC.^8^^,^^9^ Under aqueous alkaline conditions, 3-oxo-C12-HSL can either ring open to form the corresponding homoserine compound^10^ or undergo a rearrangement reaction to form 5-hydroxyethyl-3-decanoyltetramic acid (5HE-C10-TMA).^11^ 5HE-C10-TMA (denoted as C10-TA)^12^ retains both growth and quorum sensing inhibitory properties.^9^^,^^12^ It belongs to the tetramic acid (TMA) compound class that includes diverse natural products such as reutericyclin active against Gram-positive pathogens including staphylococci, clostridia and streptococci.^13–15^ The cellular targets of the antibacterial TMAs range from cell wall biosynthesis, DNA topoisomerase and RNA polymerase to membrane potential.^15^ 5HE-C10-TMA in common with reutericyclin dissipates the cytoplasmic membrane potential and pH gradient, and is an iron(III) chelator.^12^ Serial passage of *S. aureus* in sub-growth inhibitory concentrations of 5HE-C10-TMA did not select for resistance.^11^^,^^12^ Although active against planktonic bacteria, 5HE-C10-TMA showed no efficacy against *S. aureus* biofilms.^12^

Extending the C10 acyl side chain of 5HE-C10-TMA increased potency such that the MIC and *agr* IC_50_ of 5-hydroxyethyl-3-tetradecanoyltetramic acid (5HE-C14-TMA) towards *S. aureus* were 12.5 and 14 μM, respectively.^9^ Removal of the 5-hydroxyethyl moiety of 5HE-C10-TMA to generate C10-TMA did not reduce antibacterial activity.^9^ By replacing the tetramic ring nitrogen with oxygen, a series of 3-acyltetronic acids were generated that lack the iron-chelating property of 5HE-C10-TMA. Of these, 3-tetradecanoyltetronic acid (C14-TOA) was the most potent (MIC 25 μM; *agr* IC_50_ 3 μM). Both 5HE-C14-TMA and C14-TOA bound to the staphylococcal membrane with K_d_ values of 4 and 2 μM, respectively, and hyperbolic binding profiles consistent with a non-co-operative single site binding model.^9^ In a murine arthritis model neither C14-TOA nor 5HE-C10-TMA were toxic, but only C14-TOA reduced the frequency and severity of arthritis.^9^

Here, we build on our previous observations, by evaluating the antibacterial and anti-biofilm potential of 5HE-C14-TMA, C14-TOA and a novel sulphur-containing analogue (3-tetradecanoylthiotetronic acid; C14-TTA) towards MSSA and MRSA strains of *S. aureus* using a clinically relevant *in vitro* and *in vivo* model of IVC infection and examine their potential clinical use as CLSs.^9^^,^^16^

## Materials and methods

### Bacterial strains and growth conditions

Community-acquired MRSA (CA-MRSA) strain USA300, its isogenic *agr* mutant,^17^ the hospital-acquired MRSA (HA-MRSA) strain BH1CC,^18^ the HA-MSSA strain BH48^19^ and the MSSA strain SH1000^20^ were cultured in Mueller–Hinton broth for susceptibility testing or in RPMI-1640 (Gibco) for biofilm formation. For the rat jugular vein catheter infection model, the *S. aureus* strain USA300 (LAC) *lux*^21^ was used.

### Antibacterial agents and susceptibility testing

5HE-C14-TMA and C14-TOA were synthesized as described previously^9^ and C14-TTA as described in the Supplementary data available at *JAC* Online. Stock solutions of the compounds in DMSO at 10–50 mM were further diluted into tissue culture media or sodium chloride for the *in vivo* experiments. A DMSO solvent control was used in all *in vitro* experiments. The following antibiotics were used: vancomycin (Vancocin; Flynn Pharma Ltd., Republic of Ireland), daptomycin (Cubicin; Novartis, UK), gentamicin (Hospira, UK) and tigecycline (Tygacil; Pfizer Inc.). MICs were determined by broth microdilution in Mueller–Hinton broth, as recommended by the CLSI.

### Bactericidal activity

*S. aureus* USA300 LAC was grown overnight in LB medium, diluted 250-fold and grown to an OD_600_ 0.5. Cells were diluted to 1 × 10^6^ per mL in challenge medium (LB plus 5HE-C14-TMA, C14-TOA or C14-TTA at 8 × MIC). Samples were removed and viable counts determined at 30 min intervals for the first hour followed by hourly sampling. The CLSI defines a compound as bactericidal if it reduces the starting inoculum colony count by at least 99.9% (3-logs).

### Bacterial membrane permeabilization assays

Membrane permeabilization was evaluated by quantifying (i) ATP release,^22^ and (ii) the uptake of propidium iodide.^22^*S. aureus* strain USA300 LAC was grown overnight in CYGP medium,^9^ washed with sodium phosphate buffer (100 mM, pH 7), re-suspended to OD_600_ 0.1 and incubated at 37 °C with a range of concentrations of each compound for 1 h. Nisin (0.6 μM) was used as a positive control. Cell-free supernatants were assayed for ATP using the Cell Titre-Glo kit (Promega). Staphylococcal cells were re-suspended in 100 mM sodium phosphate buffer (pH 7) containing propidium iodide (10 mg/L), incubated in the dark at room temperature for 10 min. After washing, propidium iodide fluorescence (excitation: 575 nm; emission 630 nm) was determined.

### Dissipation of bacterial membrane potential

Transmembrane potential was determined as described by Breeuwer and Abee^23^ Staphylococcal cells were resuspended in potassium phosphate buffer (50 mM, pH 7) containing 5 μM of the cationic fluorescent dye DiSC_3_(5) (Invitrogen) and fluorescence determined (excitation 650 nm, emission 680 nM).

### Comparative susceptibility of S. aureus biofilms to antimicrobials

Biofilms were formed as previously described to mimic *in vivo* conditions that promote formation of coagulase-mediated biofilms.^16^ Briefly, microtitre plates or glass-bottom culture dishes (MaTek Co., USA) were coated with either 20% (v/v) or 100% (v/v) human plasma, respectively, and inoculated with *S. aureus* at an OD_600_ ∼0.2. After 1 h, non-adherent bacteria were aspirated, replaced with fresh RPMI-1640 and biofilms allowed to form over 24 h at 37 °C. Pre-formed biofilms were treated for 24 h with either 5HE-C14-TMA, C14-TOA or C14-TTA or a positive control [ethanol at 40% (v/v)]. Biofilm viability was measured by (i) resazurin-conversion assay (in microtitre wells), or (ii) live/dead imaging (glass-bottom dishes). Conversion of the non-fluorescent redox dye, resazurin into the fluorescent resorufin was performed as described previously.^16^ A 44 μM resazurin (Sigma-Aldrich, UK) solution in RPMI-1640 was added to an equal biofilm volume and incubated for 1 h at 37ºC. Fluorescence (excitation 544 nm, emission 590 nm) was quantified using a Perkin Elmer 2030 Multilabel Reader Victor^TM^ X3. Biofilms were stained with SYTO9 Green (at 13.36 μM) and propidium iodide (at 80 μM) (Molecular Probes^®^) for 1 h prior to washing. Four representative images at ×40 and ×100 were obtained per experimental group, per experiment using a Zeiss LSM 510 META confocal microscope. Each experiment was repeated at least three times. Fluorescence intensity was quantified using Image J1 software (http://imagej.net/Welcome).

### MTT cell viability assay

Human keratinocyte (HaCaT) cells were cultured in DMEM supplemented with 10% (v/v) FBS. Primary human umbilical vein endothelial cells (HUVECs) were cultured in EGM™-2 Bulletkit (Lonza) medium. HaCaT and HUVECs were harvested using TrypLE^TM^ Express (Gibco). Cells were seeded in microtitre plates at 3 × 10^5^ cells/mL and incubated for 24 h at 37 °C with an antimicrobial compound (up to 10 mg/L) or Triton X100 at 1% (v/v). After removing the medium, cells were incubated with 500 mg/L MTT (Sigma-Aldrich) for 4 h. The crystals formed were solubilized with DMSO. Absorbance was recorded at 560 nm and the CC_50_ was determined.

### Haemolysis assay

Human blood containing EDTA (1.6 g/L) was centrifuged at 1000 **g** for 5 min. Red blood cells (RBC) were washed twice with PBS, diluted 2-fold and incubated with compounds or Triton X100 (positive control) at 0.5% (v/v). *A*_570_ was determined to quantify haemolysis. IC_50_s were derived from the corresponding dose–response curves.

### Cytokine release assay

EDTA-treated human blood was incubated in microtitre wells containing either *S. aureus* USA300 biofilm, 5HE-C14-TMA (at 256 mg/L) only or biofilm with 5HE-C14-TMA (at 256 mg/L) and incubated for 2 h at 37 °C. Plasma samples were collected by centrifugation (1000 **g**) and assayed for human cytokines using a Bio-Plex 200 (Bio-Rad). A standard curve was employed to maximize sensitivity for samples containing low cytokine levels. At least three different donor blood samples (with three replicates for each) were used.

### Rat IVC infection model

Rats (male, Sprague–Dawley) were pre-implanted with jugular vein polyurethane catheters (13.5 cm long, outside diameter 1.1 mm, inside diameter 0.6 mm) (Charles River, UK). Daily flushing was performed with 150 μL sodium chloride [0.1% (w/v)] following introduction of 40 μL heparin (100 IU), sodium chloride 0.1% (w/v) within the lumen. IVC-biofilm infection was developed as previously reported.^24–26^ Catheters were inoculated with 40 μL *S. aureus* USA300*lux* suspension at 1 × 10^4^ cfu/mL ‘locked’ within the catheter for 1 day. Intravenous vancomycin (50 mg/kg) was administered twice daily at 12 h intervals to prevent systemic spread of infection. CLS consisting of 5HE-C14-TMA (40 μL) at 512 mg/L in sodium chloride 0.1% (w/v) was administered daily for 5 days upon development of infection. After 5 days of treatment, catheters were removed and luminescence imaged using a Perkin Elmer IVIS Spectrum instrument (exposure, 20 s; binning: 4, f1). Bacteria were harvested from the catheters by treatment with TrypLE^TM^ Express (Gibco) and washing prior to determining viable counts.

### Ethics approval

Ethics approvals for blood collection and use (REC820 and REC951) and animal experiments (REC931) were granted by the Ethics Committee of the Royal College of Surgeons in Ireland. *In vivo* procedures were approved by Health Products Regulatory Authority and performed under Irish Government Department of Health guidelines.

### Statistics

Statistical significance was assessed using one-way ANOVA except for Figure 6 where an unpaired t-test was applied. Statistical significance was indicated as *P < 0 .05, **P < 0.001 and ***P < 0.0001.

## Results

### Bactericidal activity of 5HE-C14-TMA, C14-TOA and C14-TTA

**Figure 1:**
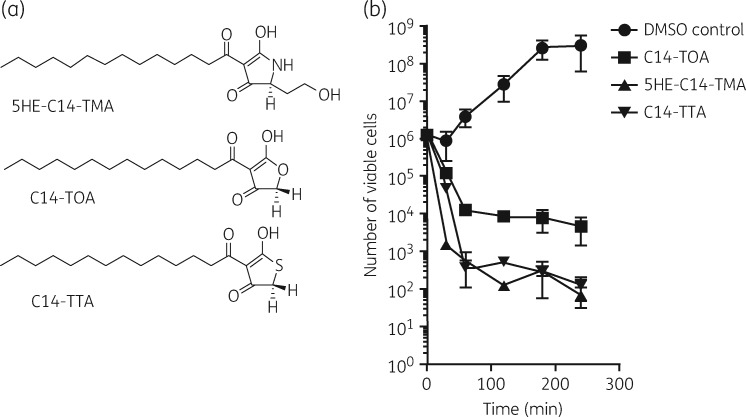
(a) Chemical structures of 5HE-C14-TMA, C14-TOA and C14-TTA. (b) Planktonic *S. aureus* USA300 cells were treated with 8 × MIC of 5HE-C14-TMA, C14-TOA or C14-TTA, respectively, or a DMSO solvent control. Data presented are the mean viable counts (cfu) per mL from three independent experiments (±SD).

5HE-C14-TMA, C14-TTA and C14-TOA (Figure 1a) each inhibited growth of MRSA (USA300 JE2 and BH1CC) and MSSA (SH1000 and BH48) strains with MICs from 2 to 4 mg/L (Table 1). 5HE-C14-TMA and C14-TTA, but not C14-TOA, were bactericidal (Figure 1b); at 8 × MIC, 5HE-C14-TMA reduced the viability of USA300 by 3 logs over 30 min.

**Table 1:** MICs of 5HE-C14-TMA, C14-TOA and C14-TTA for MRSA (USA300 and BH1CC) and MSSA (SH1000 and BH48) strains

Compound	MIC [μM (mg/L)] for *S. aureus* strain			
	USA300	BH1CC	SH1000	BH48
5HE-C14-TMA	6.25 (2)	6.25 (2)	6.25 (2)	6.25 (2)
C14-TOA	12.5 (4)	12.5 (4)	12.5 (4)	12.5 (4)
C14-TTA	12.5 (4)	12.5 (4)	12.5 (4)	12.5 (4)

### 5HE-C14-TMA, C14-TTA and C14-TOA perturb the S. aureus cytoplasmic membrane

**Figure 2:**
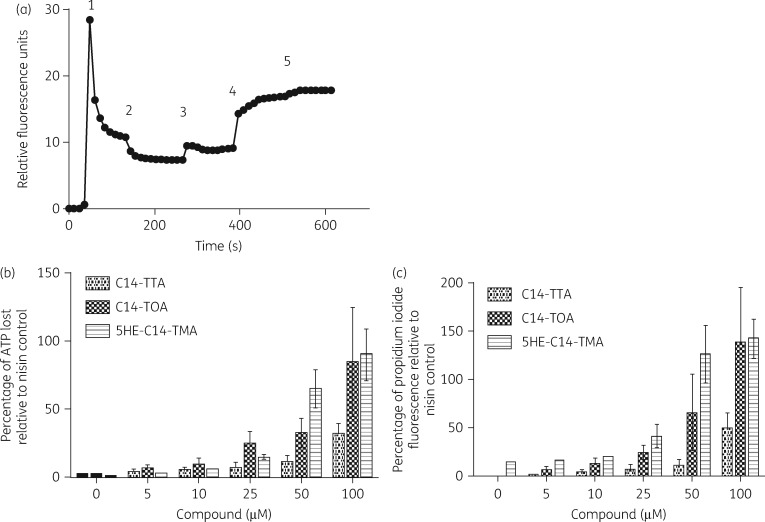
Perturbation of staphylococcal cytoplasmic membrane structure and function by 5HE-C14-TMA, C14-TTA and C14-TOA. (a) Depolarization of transmembrane potential by 5HE-C14-TMA. (1) Cationic fluorescent dye DiSC_3_(5) was added to cells followed (2) by glucose (10 mM), (3) nigericin (5 μM) to abolish the pH gradient and (4) 5HE-C14-TMA (30 μM). Complete depolarization of the membrane potential was achieved by the addition of 5 μM valinomycin (5). Experiments were carried out on three independent occasions with representative data presented. (b) Cytoplasmic ATP release relative to the positive control (nisin, 0.6 μM) of *S. aureus* cells treated with (from left to right) 0, 5, 10, 25, 50 and 100 μM 5HE-C14-TMA, C14-TTA or C14-TOA. (c) Membrane permeability. The percentage of propidium iodide uptake relative to the positive control (nisin, 0.6 μM) of *S. aureus* cells treated with (from left to right) 0, 5, 10, 25, 50 and 100 μM 5HE-C14-TMA, C14-TTA or C14-TOA. Data plotted are the mean values of three independent experiments; error bars represent standard deviations.

5HE-C14-TMA and C14-TOA (a tetronic acid analogue lacking the 5-hydroxyethyl substituent) both bind to *S. aureus* membranes with high affinity.^9^ Lowery *et al.* showed that 5HE-C10-TMA could dissipate the cytoplasmic transmembrane potential (ΔΨ).^12^ To determine whether 5HE-C14-TMA, C14-TTA and C14-TOA exhibit similar modes of action, the change in membrane potential following exposure to each compound was determined using the cationic fluorescent dye DiSC_3_(5). 5HE-C14-TMA (30 μM) caused an increase in fluorescence, indicative of membrane depolarization (Figure 2a). Similar results were obtained for C14-TTA and C14-TOA (Figure S1, available as Supplementary data at *JAC* Online). To investigate the extent of membrane perturbation caused by 5HE-C14TMA and related compounds, the uptake of propidium iodide in conjunction with the loss of the cytoplasmic ATP was assessed. Figure 2(b and c) shows that propidium iodide fluorescence and extracellular ATP levels increased with increasing concentrations of each compound, suggesting loss of membrane integrity is the likely cause of bacterial cell death. For both assays, C14-TTA was the least active.

### Biofilm killing activity

**Figure 3:**
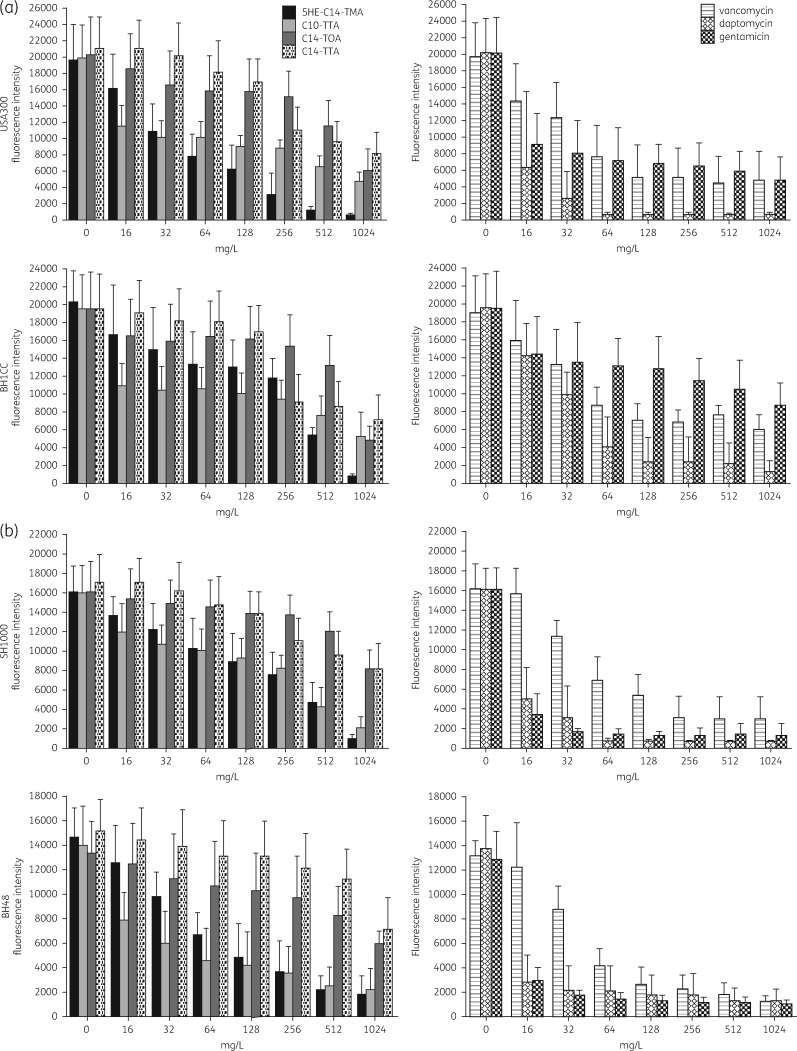
Comparative dose-dependent killing of biofilms formed by (a) MRSA strains USA300 Lac and BH1CC and (b) MSSA strains SH1000 and BH48 by 5HE-C14-TMA, C14-TTA, C14-TOA or the antibiotics vancomycin, daptomycin or gentamicin. *S. aureus* biofilms were grown in plasma-coated microtitre plate wells in RPMI-1640 for 24 h at 37 °C. 5HE-C14-TMA, C14-TOA or C14-TTA (or antibiotics) were incubated in the wells at a range of concentrations for 24 h. Resazurin-conversion assay was used to assess biofilm viability. Mean fluorescence intensities (±SD) of three independent experiments are shown.

**Figure 4:**
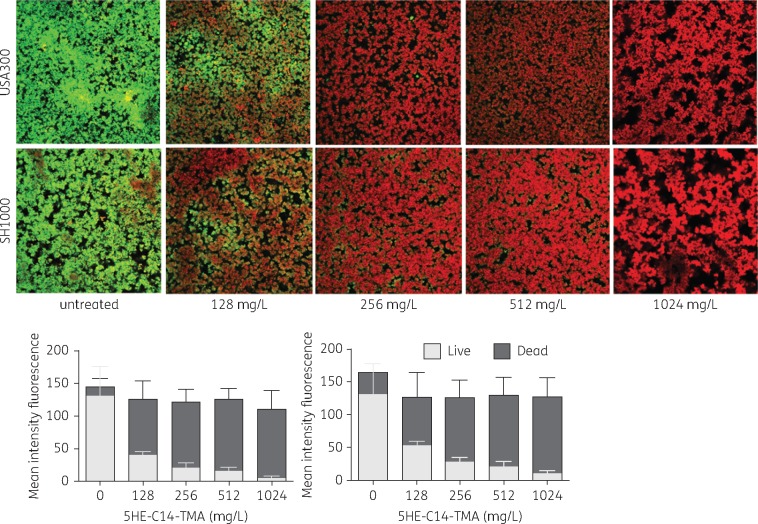
Dose-dependent killing of *S. aureus* USA300 and SH1000 biofilms by 5HE-C14-TMA. Bacterial biofilms were grown in plasma-coated glass-bottom MaTeK^TM^ dishes in RPMI-1640 for 24 h at 37 °C. 5HE-C14-TMA was added at a range of concentrations and incubated for a further 24 h. Biofilm bacterial viability was evaluated using live/dead imaging (top panels), quantified using ImageJ and presented as mean fluorescence intensity (±SD) (left histogram, USA300; right histogram, SH1000).

The biofilm-killing activity of 5HE-C14-TMA, C14-TOA and C14-TTA against *in vivo-*relevant biofilms of *S. aureus* was compared with clinically relevant antibiotics. Although all three compounds reduced biofilm viability, C14-TOA and C14-TTA were only active at the highest concentrations (512 and 1024 mg/L) reducing viability by ∼2.5–3-fold (Figure 3). In contrast, 5HE-C14-TMA (at 1024 mg/L) effectively eradicated biofilm reducing viability by ∼7–20-fold depending on the strain (Figure 3). This activity profile was similar to that of vancomycin although the latter only reduced MRSA biofilm viability by ∼3.5–4-fold at 1024 mg/mL (Figure 3). In contrast, daptomycin was the most active antimicrobial evaluated (Figure 3). The *in vitro* activity of 5HE-C14-TMA was further validated using live/dead staining. Figure 4 confirms the dose-dependent loss of viability of *S. aureus* USA300 and SH1000 biofilms and shows that substantial killing (>50%) occurs with as little as 128 mg/L for both strains.

### Cytotoxicity and haemolytic activity

To obtain comparative cytotoxicity data for 5HE-C14-TMA, C14-TOA and C14-TTA, we examined the responses of HaCaT keratinocytes and primary HUVEC cells using the MTT viability assay. Table 2 shows that C14-TTA (CC_50_ 26 μM) is more cytotoxic for HaCaT cells than 5HE-C14-TMA (CC_50_ 181 μM) or C14-TOA (CC_50_ 257 μM). For HUVECs, 5HE-C14-TMA and C14-TOA showed similar CC_50_s (Table 2). In contrast, C14-TOA was far less haemolytic towards human RBC (CC_50_ >2048 μM/>667 mg/L) than C14-TTA or 5HE-C14-TMA (Table 2). The ‘selectivity index’ (SI) of a given agent provides a measure of the preference for bacterial rather than mammalian cells and is defined as the ratio of mammalian cell cytotoxicity to MIC (CC_50_/MIC).^27^ Compounds with SI values >10 are usually considered suitable for *in vivo* evaluation. For HaCaT cells, SI values of 32, 20 and 2, respectively, were obtained for 5HE-C14-TMA, C14-TOA and C14-TTA suggesting that 5HE-C14-TMA shows the greatest selectivity. 5HE-C14-TMA and C14-TOA showed less selective toxicity towards HUVECs with SIs 3.5 and 2.3, respectively.

**Table 2:** Cytotoxic and haemolytic activities of 5HE-C14-TMA, C14-TOA and C14-TTA

Cell type	CC_50_ [μM (mg/L)]		
	5HE-C14-TMA	C14-TOA	C14-TTA
HaCaT	181 (64)	257 (80)	26 (8.3)
HUVEC	19 (7)	28 (9)	not determined
RBC	246 (87)	>2048 (>667)	470 (153)

### 5HE-C14-TMA treatment reduces release of pro-inflammatory cytokines

**Figure 5:**
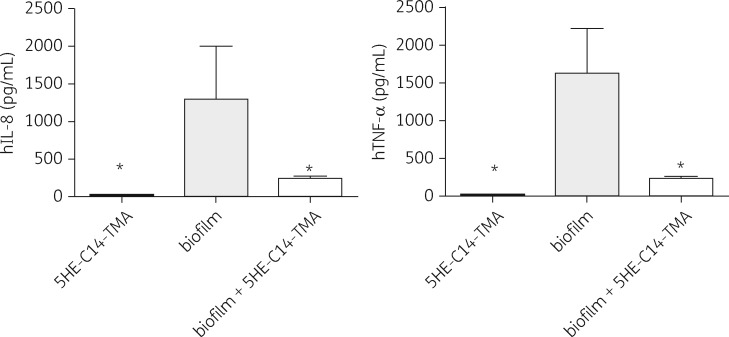
Human IL-8 and TNF-α concentrations in whole human blood following exposure to 5HE-C14-TMA (256 mg/L), untreated USA300 biofilm or USA300 biofilm treated with 5HE-C14-TMA (256 mg/L) for 2 h. Supernatants were collected and the plasma fraction subjected to a Bio-Plex Precision Pro™ (magnetic) human cytokine immunoassay (Bio-Rad). Means of cytokine concentrations obtained from three donors (±SD) are shown. Statistical significance was determined using one-way ANOVA and is indicated (*P <* *0.5). h, human.

Cytokine levels in whole human blood were quantified upon exposure to *S. aureus* USA300 biofilm, 5HE-C14-TMA, or the biofilm with 5HE-C14-TMA.^16^ The levels of the pro-inflammatory IL-8 and TNF-α were significantly elevated in the blood exposed to biofilm while IL-2, IL-4, IL-6, IL-10, GM-CSF and IFN-γ remained unchanged (Figure 5 and data not shown). There was no increase in cytokine levels for the sample containing the agent only. However, exposure of the same biofilm to a sub-bactericidal dose (256 mg/L for 2 h) of 5HE-C14-TMA resulted in substantial reductions in IL-8 and TNF-α. These data suggest that 5HE-C14-TMA modifies the immunogenicity of *S. aureus* biofilms (Figure 5). Given that 5HE-C14-TMA inhibits the *S. aureus agr* quorum sensing system, we examined the cytokine levels produced in response to an *S. aureus* USA300 *agr* mutant biofilm treated with or without 5HE-C14-TMA. The data presented in Figure S2 show that, while both IL-8 and TNF-α are significantly reduced in response to the *agr* mutant biofilm, the same dose of 5HE-C14-TMA had no further effect on the levels of the two cytokines.

### In vivo activity of 5HE-C14-TMA against S. aureus biofilm

**Figure 6:**
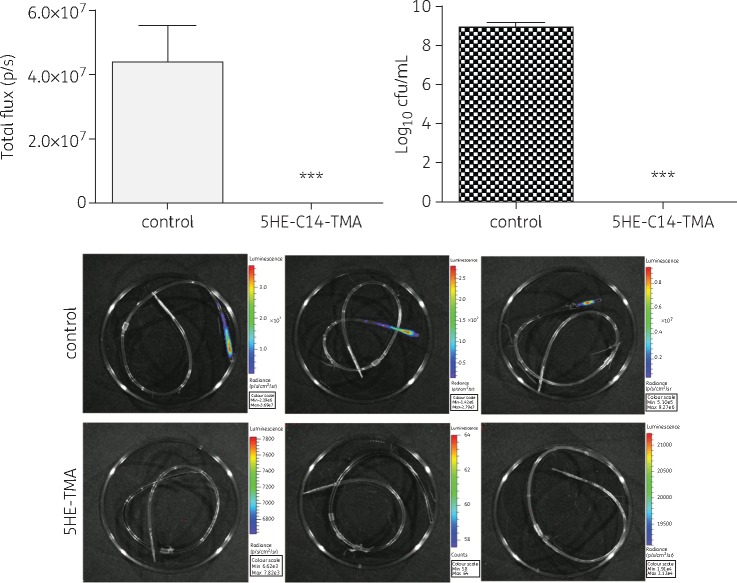
*In vivo* eradication of an MRSA biofilm with 5HE-C14-TMA. CLS consisting of 5HE-C14-TMA (512 mg/L) was instilled into a jugular vein catheter to treat IVC-associated *S. aureus* USA300*lux* biofilms. In the control group the CLS was replaced with 0.9% sodium chloride. CLS was renewed every 24 h for 5 days. The day after the final treatment, animals were sacrificed, catheters removed and subjected to quantification of bioluminescence using IVIS. Bacterial cells were harvested using TrypLE^TM^ reagent, serially diluted and plated on tryptic soy agar for cfu counting. Statistical significance is indicated (***P <* *0.001). This figure appears in colour in the online version of *JAC* and in black and white in the print version of *JAC*.

The efficacy of 5HE-C14-TMA as a CLS in an *in vivo* rat model of IVC infection using the MRSA strain USA300*lux* was investigated (Figure 6). Animals treated with CLS containing sodium chloride [0.1% (w/v)] were colonized by ∼10^9^ cfu/mL at day 5 of the treatment regimen while in catheters treated with 5HE-C14-TMA the biofilm was effectively eradicated, suggesting *in vivo* efficacy of the compound.

## Discussion

An important treatment option for the salvage of an IVC in patients with a CRBSI due to *S. aureus* is the use of a CLS in combination with systemic antibiotics.^5^ In choosing an antimicrobial agent for the CLS, consideration must be given to the likely effectiveness of the agent against *S. aureus* biofilms in the context of the environmental conditions in which such biofilm is formed.^6^ We recently developed an *in vitro* biofilm model of IVC infection employing physiologically relevant iron-deficient RPMI-1640 medium in conjunction with human plasma-conditioned surfaces which generate coagulase-mediated and fibrin-embedded biofilms that mimic those formed *in vivo*. These biofilms, unlike those formed in rich laboratory media under standard conditions demonstrated antibiotic susceptibilities similar to those found for *in vivo* infections established by *S. aureus* biofilms.^6^^,^^16^ Given the need for novel and effective agents for the treatment of device-related *S. aureus* infections that could be used as CLS or as potential treatment options for other device-related infections, we explored the mode of action and inhibitory activity of compounds that are related to a natural product (5HE-C10-TMA).^11^ This natural product is formed *in vivo* during chronic *P. aeruginosa* lung infections in individuals with cystic fibrosis and may well contribute to the eradication of competitor bacteria including *S. aureus.*^12^^,^^28^ Although 5HE-C10-TMA inhibits the growth of planktonic Gram-positive bacteria, it was reported to lack activity against *S. aureus* biofilms formed in rich laboratory media on polystyrene.^9^^,^^12^^,^^29^ Similarly, 5HE-C10-TMA was inactive in a murine arthritis infection model.^9^ Consequently we synthesized a series of TMAs and related compounds to provide a structure–function relationship (SAR) and to identify compounds with greater antibacterial and anti-biofilm activity.^9^

The most active TMA identified in our SAR study was the C14 analogue of 5HE-C10-TMA that exhibited an 8-fold increase in growth inhibitory activity against planktonic *S. aureus.*^9^ These data are also consistent with a reutericyclin SAR study that revealed that lipophilic analogues were generally more active against Gram-positive pathogens than those containing polar and charged substituents.^15^ Since ferric iron was reported to abolish the antibacterial activity of 5HE-C10-TMA towards *Clostridium difficile*^29^ we also synthesized variants of the TMAs in which we extended the acyl chain and replaced the ring nitrogen with oxygen or sulphur to generate the C14 tetronic (TOA)^9^ and thiotetronic (TTA; this paper) acids that in contrast to the TMAs, do not chelate iron (data not shown). The MICs of C14-TOA and C14-TTA at 4 mg/L were marginally higher than 5HE-C14-TMA (2 mg/L) for the MRSA and MSSA strains investigated. However, only 5HE-C14-TMA and C14-TTA were bactericidal in time-dependent killing assays at 8 × MIC against the CA-MRSA strain USA300 LAC. Consistent with their ability to bind to the *S. aureus* membrane with high affinity, 5HE-C14-TMA, C14-TOA and C14-TTA in common with 5HE-C10-TMA and antibiotics such as reutericyclin, daptomycin and telavancin dissipate the transmembrane potential, increase cellular permeability and promote the release of ATP.^9^^,^^12^^,^^22^^,^^30^ For 5HE-C10-TMA, dissipation of the transmembrane potential and pH gradient rather than membrane permeabilization were suggested to correlate most closely with bacterial cell death.

5HE-C10-TMA and 5HE-C12-TMA have been suggested to be non-toxic to human cells since neither reduced the viability of bone marrow-derived macrophages up to 100 μM.^12^ However, we observed that the cytotoxicity of 5HE-C14-TMA, C14-TOA and C14-TTA was cell type dependent. For example, 5HE-C14-TMA reduced the viability of HUVEC cells at much lower concentrations than for HaCaT cells. Nevertheless the SIs for 5HE-C14-TMA indicates that this compound is more selective for bacteria than mammalian cells.

Although 5HE-C10-TMA was inactive at 200 mg/L against *S. aureus* biofilms,^12^ we observed that the longer chain analogue, 5HE-C14-TMA killed over 50% of both MRSA and MSSA biofilms at 128 mg/L with complete killing achieved at 512–1024 mg/L. However, the differences observed may not only reflect the increased anti-biofilm activity of 5HE-C14-TMA compared with 5HE-C10-TMA, achieved via the four carbon acyl chain extension, but also the nature of the biofilm model used as a more *in-vivo*-like model of biofilm infection was used in this study. In contrast to 5HE-C14-TMA, and despite the similarities with respect to MIC and mechanism of action, C14-TOA and C14-TTA were far less effective at reducing the viability of either MSSA or MRSA biofilms. Since 5HE-C14-TMA, in contrast to C14-TOA and C14-TTA, is an iron chelator and as iron availability impacts on *S. aureus* biofilm development,^31^ the differential anti-biofilm activity observed may be due in part to this additional functionality.

The reduced anti-biofilm activity of C14-TOA compared with 5HE-C14-TMA is interesting given that C14-TOA significantly reduced the frequency and severity of arthritis and joint destruction in the same murine *S. aureus* infection model in which 5HE-C10-TMA was inactive.^9^ C14-TOA did not, however, reduce the numbers of viable staphylococci in the kidneys suggesting that the anti-virulence properties of the molecule as an *agr* inhibitor may account for the positive outcome on arthritis observed. Although 5HE-C14-TMA is a less effective *agr* inhibitor than C14-TOA,^9^*S. aureus* biofilms treated with sub-growth inhibitory 5HE-C14-TMA did not elicit production of IL-8 and TNF-α when exposed to human blood. This was also the case for *agr* mutant biofilms suggesting that 5HE-C14-TMA modifies the immunogenicity of *S. aureus* biofilms by inhibiting the production and release of *agr*-dependent pro-inflammatory toxins.^32^

Encouragingly, 5HE-C14-TMA was effective in the eradication of MRSA and MSSA biofilms formed *in vitro*, but under *in vivo*-mimicking conditions. To extend our *in vitro* findings a rat model of IVC infection assessed the effectiveness of 5HE-C14-TMA *in vivo*. 5HE-C14-TMA was highly effective in the eradication of MRSA biofilm formed in this model, whilst untreated catheters resulted in harvesting of 10^9^ cfu/mL biofilm.

Taken together, the *in vitro* and *in vivo* efficacy of 5HE-C14-TMA towards *S. aureus* biofilms suggests that this compound scaffold has therapeutic potential for the treatment of *S. aureus* IVC infections within a CLS.

## Supplementary Material

Supplementary Data
