# Supplementary material for: 5-Hydroxyethyl-3-tetradecanoyltetramic acid represents a novel treatment for intravascular catheter infections due to Staphylococcus aureus
Source: J Antimicrob Chemother. 2016 Dec 19;72(3):744–53. doi: 10.1093/jac/dkw482 (PMC5400099; doi:10.1093/jac/dkw482)

**Supplementary data**

***Synthesis of 3-Tetradecanoylthiotetronic acid (C14-TTA)***

The chemical synthesis of 3-tetradecanoylthiotetronic acid (C14-TTA) was accomplished in 5 steps as shown in **Scheme 1**. Thioglycolic acid was acetylated with acetic anhydride and the *S*-acetylated product was condensed with Meldrum’s acid using water soluble carbodiimide to give 5-acylated Meldrum’s acid as a orange brown solid. The latter when refluxed in methanol gave a β-keto ester, methyl 4-(acetylthio)-3-oxobutanoate as a brown oil. The β-keto ester was then acylated with tetradecanoyl chloride using sodium hydride to afford methyl 2-[2-(acetylthio)acetyl]-3-oxohexadecanoate as a yellow solid after chromatographic purification. The yellow solid when dissolved in a solution of sodium hydroxide and stirred overnight underwent *S*-deacetylation and subsequent cyclization to deliver C14-TTA as a purple solid after purification.

***2-(Acetylthio)acetic acid***

Acetic anhydride (3.24 g/3 mL, 31.7 mmol) was added to a solution of thioglycolic acid (2.64 g≡2 mL, 28.7 mmol) in acetonitrile (20 mL) containing triethylamine (5.81 g≡8 mL, 57 mmol) and 4-dimethylaminopyridine (0.01 g) at 0^o^C. The mixture was stirred overnight at room temperature. The solvent was removed under reduced pressure and the resulting residue was taken up in 1 M HCl (30 mL) and extracted with ethyl acetate (2x 25 mL). The combined organic layer was washed with water (20 mL) and brine (20 mL), dried over magnesium sulphate and concentrated to give a clear oil which was then dried in a desiccator to remove traces of acetic acid (2.712 g, 70.4%).

^1^H NMR (CDCl3) δ 2.45 (3H, s, Me), 3.76 (2H, s, CH_2_)

***S-(2-(2,2-Dimethyl-4,6-dioxo-1,3-dioxan-5-yl)-2-oxoethyl) ethanethioate***

A mixture of 2-(acetylthio)acetic acid (2.709 g, 20.2 mmol), 4-dimethylaminopyridine (3.69 g, 30.3 mmol) and Meldrum’s acid (3.05 g, 21.2 mmol) in dry dichloromethane (40mL) was stirred at room temperature. 1-ethyl-3-(3-dimethylaminopropyl)carbodiimide.HCl (4.26 g, 22.2 mmol) and triethylamine (2.04 g≡2.81 mL, 20.2 mmol) were added and the mixture was stirred overnight at room temperature. The solution was then washed with 1 M HCl (2x 30 mL) and brine (20 mL). The organic layer was dried over magnesium sulphate and concentrated to give an orange brown solid. (4.291 g, 81.6%)

^1^H NMR (CDCl3) δ 1.81 (6H, s, Me x 2), 2.45 (3H, s, Me), 3.76 (2H, s, CH_2_)

***Methyl 4-(acetylthio)-3-oxobutanoate***

A solution of *S*-(2-(2,2-Dimethyl-4,6-dioxo-1,3-dioxan-5-yl)-2-oxoethyl) ethanethioate (4.288 g) and dry methanol (40 mL) was stirred at reflux for 2 h. The mixture was cooled and concentrated, dissolved in ethyl acetate (50 mL), then washed with saturated sodium bicarbonate (25 mL), 1 M HCl (25 mL), and brine (25 mL). The organic layer was dried over magnesium sulphate and concentrated to give a brown oil. (2.275 g, 72.8%)

^1^H NMR (CDCl_3_) δ 2.40 (3H, s, Me), 3.63 (2H, s, COCH_2_CO), 3.78 (3H, s, OMe), 3.88 (2H, s, CH_2_S)

***Methyl 2-(2-(acetylthio)acetyl)-3-oxohexadecanoate***

A solution of methyl 4-(acetylthio)-3-oxobutanoate (0.065 g, 3.5 mmol) in dry toluene (4 mL) was added to a slurry of sodium hydride [60% dispersion in mineral oil] (0.154 g, 3.85 mmol) at room temperature over 15 min. The mixture was stirred at room temperature for 1 h and then a solution of tetradecanoyl chloride (0.684 g≡0.951 mL, 3.5 mmol) in dry toluene (4 mL) was added over 10 min. The stirring was continued at room temperature for 24 h. The mixture was then washed with water (2x 10 mL), 1 M HCl (2x 10 mL), and brine (10 mL).The organic layer was dried over magnesium sulphate and concentrated to give an oily brown solid which was purified using Flash chromatography (5% ethyl acetate in hexane) to deliver the title compound as a yellow solid (0.406 g, 29%)

^1^H NMR (CDCl_3_) δ 0.92 (3H, t, Me), 1.28-1.34 (20H, m, *(CH_2_)_10_*Me), 1.66 (2H, m, *CH_2_*(CH_2_)_10_Me), 2.40 (3H, s, COMe), 2.69 (2H, t, *CH_2_*(CH_2_)_11_Me), 4.08 (3H, s, OMe), 4.15 (2H, s, CH_2_S)

***3-Tetradecanoylthiotetronic acid***

A solution of sodium hydroxide (0.08 g, 2 mmol) in water (2.0 mL) was added to methyl 2-(2-(acetylthio)acetyl)-3-oxohexadecanoate (0.402 g, 1.0 mmol) in THF (5 mL) and stirred overnight at room temperature. The THF was removed under vacuum, acidified the residue with 1 M HCl (4 mL) and extracted into ethyl acetate (2x 15 mL). The organic layer was washed with brine (10 mL), dried over magnesium sulphate and concentrated to give a brown solid which was purified using Flash chromatography (80% ethyl acetate in hexane) to deliver the title compound as a purple solid. (0.061 g, 18.8%)

^1^H NMR (DMSO) δ 0.86 (3H, t, Me), 1.24-1.30 (20H, m, *(CH_2_)_10_*Me), 1.41 (2H, m, *CH_2_*(CH_2_)_10_Me), 2.68 (2H, s, *CH_2_*(CH_2_)_11_Me), 3.44 & 3.70 (2H, s x2)

ES-MS *m/z* 325.1839 [M-H]^-^, C_18_H_29_O_3_S requires 325.1843.

**Figure S1.** Perturbation of staphylococcal cytoplasmic membrane structure Depolarisation of transmembrane potential by (**a**) C14-TOA and (**b**) C14-TTA. (1) The cationic fluorescent dye DiSC3(5) was added to cells followed (2) by glucose (10 mM), (3) nigericin (5 µM) to abolish the pH gradient and (4) either C14-TOA or C14-TTA (30 µM). Complete depolarisation of the membrane potential was achieved by the addition of 5 µM valinomycin (5). Experiments were carried out on 3 independent occasions with representative data presented.





(a)

(b)





**Figure S2.** Human IL-8 and TNFα concentrations in whole human blood following exposure of coagulase-dependent biofilms of the *S. aureus* USA 300 wild type and isogenic *agr* mutant to 5HE-C14-TMA (256 µg/mL) or solvent control. The means of cytokine concentrations obtained for three donors ± SDs are shown. Statistical significance was determined using one-way ANOVA.


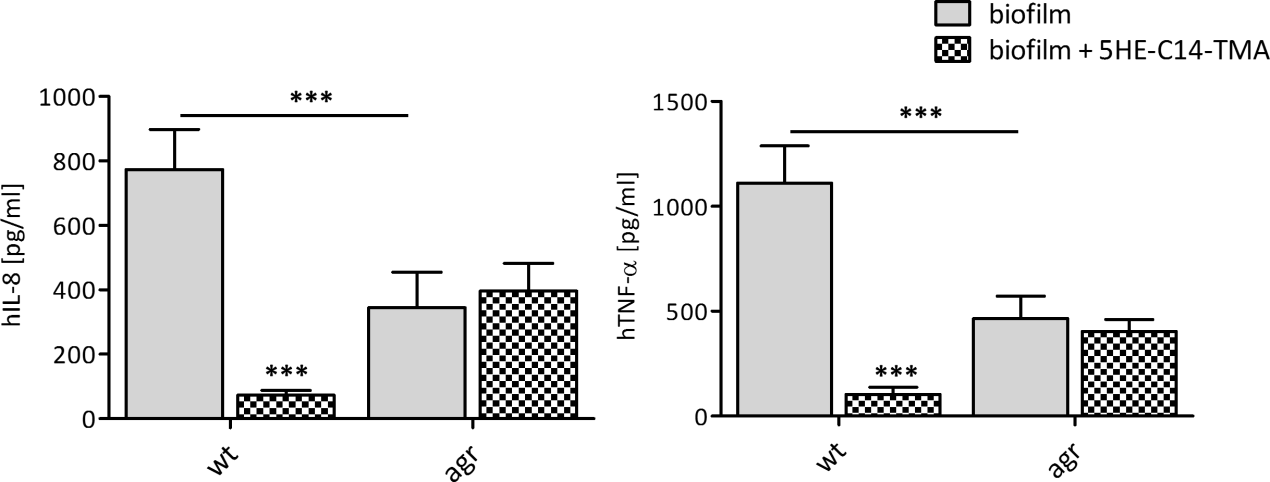

Supplement: Supplementary Data [file dkw482_Supplementary_Data.zip › Supplementary_Data.docx]
